# Smart-Home Innovation for Older Adults’ Mental Health: Reimagining Usability and Accessibility

**DOI:** 10.5888/pcd22.250113

**Published:** 2025-07-17

**Authors:** Mohammad Mahdi Fakhimi

**Affiliations:** 1 College of Design, University of Minnesota, Minneapolis, Minnesota

## Introduction

The aging population faces escalating mental health challenges, including depression, anxiety, and cognitive decline (1–3). According to the World Health Organization, the number of people aged 60 years or older will exceed 2 billion worldwide by 2050, increasing the prevalence of mental health issues in this population (1,2). Although early diagnosis and care for conditions such as late-life depression or mild cognitive impairment can prevent worsening outcomes, solutions for ongoing, in-home support for this demographic group remain limited.

Mental health disorders often present differently in older than in younger people (2,4). Late-life depression, anxiety, and cognitive impairment are frequently underdiagnosed or misattributed to aging itself (2,3). Conditions such as apathy, irritability, insomnia, and memory lapses are often overlooked despite their significant effect on quality of life (2,3). Moreover, comorbidities and social isolation exacerbate these symptoms, creating a complex interplay between psychological and physical health (2). Understanding this unique psychopathology is essential for designing systems that can sensitively detect and respond to early warning signs (2,4).

Historically, older adults have relied on clinical services, family caregivers, and community programs for mental health support (5). Although these resources are crucial, they often fail to provide real-time monitoring and timely interventions in day-to-day settings (5). In recent years, smart-home technologies have emerged as a potential alternative to traditional support systems; however, limited usability, accessibility barriers, and privacy concerns have hindered their widespread adoption (6). This essay examines why the mental health of older adults remains a major public health challenge, reviews historical approaches to care, and proposes ways to make smart-home technologies inclusive and effective.

## Why Mental Health Among Seniors is a Public Health Challenge

Older adults’ mental health has become a public health priority because of its deep social and economic consequences (2). Untreated issues compound physical conditions, limit independence, and undermine overall quality of life (2). Because many older adults prefer aging in place, domestic systems that can detect early signs of distress and offer interventions outside of crisis situations are urgently needed (7). Resource constraints, such as a limited number of home health care providers and the financial burden of frequent clinic visits, underscore the urgency for cost-efficient, continuous care. Although smart-home technologies show promise, they are often developed without full consideration of older adults’ physical, cognitive, or cultural needs, creating unintended barriers to their adoption (5).

## Historical Approaches to Mental Health Support

Traditionally, older adults turned to pharmacotherapy, psychotherapy, and family caregiving (5). In recent decades, telehealth increased access to care through telephone or video consultations; however, these interactions remained bound by scheduled appointments (6). Wearables and fall-detection alarms have been widely adopted but primarily address physical risks, leaving emotional well-being and cognitive changes as secondary concerns (8). Early-generation smart-home prototypes integrated sensors and artificial intelligence analytics to detect subtle behavioral changes, such as disrupted sleep or dietary patterns that might correlate with mood disorders, but concentrated on physical safety rather than framing mental health as a holistic element of care (9). Consequently, mood fluctuations, anxiety, or cognitive decline often remain undermonitored and underserved.

## Barriers and Proposed Framework

Despite growing evidence that well-designed smart-home technologies can bolster mental health (5,8), barriers such as poor usability, limited accessibility, and low trust persist. A human-centered, integrative framework can mitigate these issues:

**Participatory co-design**. Continuous involvement of older adults uncovers hidden usability failures and ensures cultural fit (8).
**Universal design for accessibility**. Features such as large buttons, voice control, and adjustable text size reduce cognitive load and support sensory or mobility limitations (5).
**Privacy and ethical data practices**. Transparent policies, encryption, and user-controlled permissions alleviate perceptions of surveillance (10).
**Cost and coverage expansion**. Partnerships with insurers or government subsidies can lower financial hurdles and broaden access, particularly in rural areas (7).
**Early intervention via behavioral monitoring**. Tracking sleep patterns, social withdrawal, or speech changes enables proactive prompts or alerts before mental health deteriorates (6).

**Figure:**
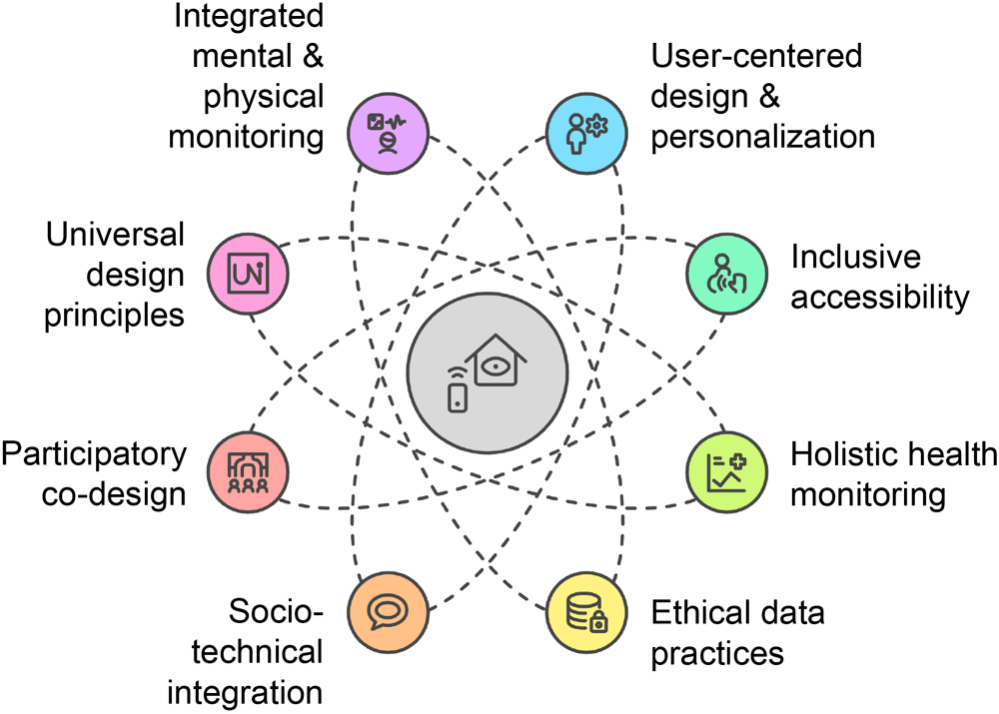
Conceptual framework linking human-centered design principles with smart-home strategies for early detection and intervention in older-adult mental health.

Everyone can benefit from smart-home design:

**Older adults**, who achieve greater independence, reduced stigma, and earlier support for mental health concerns
**Family caregivers**, who receive real-time notifications that enable faster responses to potential crises
**Health care providers**, who receive timely alerts and integrated data that may reduce urgent hospital visits
**Public health practitioners**, because supporting aging in place may lessen the social and economic burden of untreated mental health conditions

## Future Impact

With collaborative efforts, smart-home technologies could become a vital resource for older adults. Smart sensors might detect emerging signs of depression or cognitive decline well before clinical appointments. Artificial intelligence–driven platforms could connect family and health care providers, enabling remote interventions that avert crises. Automated social engagement prompts and culturally adaptive interfaces may also mitigate isolation and loneliness. When executed carefully, such technologies can promote dignity, safety, and well-being for older adults aging at home.
